# Expressing Constitutively Active Rheb in Adult Dorsal Root Ganglion Neurons Enhances the Integration of Sensory Axons that Regenerate Across a Chondroitinase-Treated Dorsal Root Entry Zone Following Dorsal Root Crush

**DOI:** 10.3389/fnmol.2016.00049

**Published:** 2016-07-05

**Authors:** Di Wu, Michelle C. Klaw, Nikolai Kholodilov, Robert E. Burke, Megan R. Detloff, Marie-Pascale Côté, Veronica J. Tom

**Affiliations:** ^1^Department of Neurobiology and Anatomy, Drexel University College of MedicinePhiladelphia, PA, USA; ^2^Department of Neurology, Columbia University in the City of New YorkNew York, NY, USA; ^3^Department of Pathology and Cell Biology, Columbia University in the City of New YorkNew York, NY, USA

**Keywords:** dorsal root crush, axon regeneration, Rheb, chondroitinase, *c-fos*, inflammation

## Abstract

While the peripheral branch of dorsal root ganglion neurons (DRG) can successfully regenerate after injury, lesioned central branch axons fail to regrow across the dorsal root entry zone (DREZ), the interface between the dorsal root and the spinal cord. This lack of regeneration is due to the limited regenerative capacity of adult sensory axons and the growth-inhibitory environment at the DREZ, which is similar to that found in the glial scar after a central nervous system (CNS) injury. We hypothesized that transduction of adult DRG neurons using adeno-associated virus (AAV) to express a constitutively-active form of the GTPase Rheb (caRheb) will increase their intrinsic growth potential after a dorsal root crush. Additionally, we posited that if we combined that approach with digestion of upregulated chondroitin sulfate proteoglycans (CSPG) at the DREZ with chondroitinase ABC (ChABC), we would promote regeneration of sensory axons across the DREZ into the spinal cord. We first assessed if this strategy promotes neuritic growth in an *in vitro* model of the glial scar containing CSPG. ChABC allowed for some regeneration across the once potently inhibitory substrate. Combining ChABC treatment with expression of caRheb in DRG significantly improved this growth. We then determined if this combination strategy also enhanced regeneration through the DREZ after dorsal root crush in adult rats *in vivo*. After unilaterally crushing C4-T1 dorsal roots, we injected AAV5-caRheb or AAV5-GFP into the ipsilateral C5-C8 DRGs. ChABC or PBS was injected into the ipsilateral dorsal horn at C5-C8 to digest CSPG, for a total of four animal groups (caRheb + ChABC, caRheb + PBS, GFP + ChABC, GFP + PBS). Regeneration was rarely observed in PBS-treated animals, whereas short-distance regrowth across the DREZ was observed in ChABC-treated animals. No difference in axon number or length between the ChABC groups was observed, which may be related to intraganglionic inflammation induced by the injection. ChABC-mediated regeneration is functional, as stimulation of ipsilateral median and ulnar nerves induced neuronal c-Fos expression in deafferented dorsal horn in both ChABC groups. Interestingly, caRheb + ChABC animals had significantly more c-Fos^+^ nuclei indicating that caRheb expression in DRGs promoted functional synaptogenesis of their axons that regenerated beyond a ChABC-treated DREZ.

## Introduction

Dorsal root ganglia (DRG) neurons have long been exploited to study axon regeneration because they feature both peripheral and central axon branches (Devor, 1999; Mar et al., 2015). While the peripheral axon can successfully regenerate after injury, lesion of the central branch, such as after trauma to the dorsal columns tract within the spinal cord or a dorsal root, often results in permanent sensory deficits (Qiu et al., 2005; Wang et al., 2008). Interestingly, after a dorsal root injury, the centrally projecting axons attempt to regenerate as long as the root is contiguous, allowing for the alignment of Schwann cells upon which these axons extend. However, this advancement ceases when the growing axon tip reaches the dorsal root entry zone (DREZ), the interface between the peripheral nervous system (PNS) and the central nervous system (CNS). The failure of sensory axon regeneration after dorsal root injury is partly attributed to a CNS environment that is not favorable for growth (Zhang et al., 2001; Mar et al., 2015). After injury, the CNS is rich in growth inhibitory proteins including Nogo, myelin-associated glycoprotein and chondroitin sulfate proteoglycans (CSPG) and lacks trophic support (Fraher, 2000; Zhang et al., 2001; Silver and Miller, 2004). Thus, at the DREZ, the transition from a permissive PNS environment to a hostile CNS one results in growth cone collapse and stalling (Fraher, 2000; Woolf and Bloechlinger, 2002; Di Maio et al., 2011). Additionally, limited intrinsic regenerative capacity of adult sensory axons also contributes to the failure of axon regeneration (Verma et al., 2005; Mar et al., 2014). Peripheral injury triggers the expression of regeneration-associated genes, such as activating transcription factor 3 (ATF-3) and growth associated protein 43 (GAP-43) whereas dorsal root injury fails to elicit a similar response (Schreyer and Skene, 1993; Seijffers et al., 2006).

While strategies aimed at overcoming either intrinsic or extrinsic obstacles of axon regeneration have shown some success in boosting axonal regrowth (Wang et al., 2008; Peng et al., 2010; Carmel et al., 2015), stimulation of the intrinsic capacity for axonal extension coupled with decreasing extrinsic barriers generates even more regeneration. For instance, activating macrophages in the eye switches mature retinal ganglion cells (RGCs) into an active growth state in an optic nerve crush injury model. When macrophage activation is combined with RhoA inactivation or Nogo receptor suppression, further axon regeneration across the lesion site was observed (Fischer et al., 2004a,b). Moreover, when zymosan-triggered inflammation to enhance the intrinsic growth capacity was combined with use of the bacterial enzyme chondrotinase ABC (ChABC) to digest inhibitory CSPG, substantially more axon regeneration across the harsh environment of DREZ was observed (Steinmetz et al., 2005).

Previously, we showed that adeno-associated virus (AAV) transduction of adult neurons with constitutively active Rheb (caRheb; Ras homolog enriched in brain) enhances regeneration of descending axons across a ChABC-treated glial scar after spinal cord injury (Wu et al., 2015). Rheb is directly upstream of mTOR (Durán and Hall, 2012). It is active when bound to GTP and is inactive when bound to GDP. caRheb is a mutated form of Rheb that is persistently bound to GTP, resulting in continual activation of mTOR (Kim et al., 2011, 2012). Increasing mTOR activity in adult neurons is sufficient to augment axonal growth from different neuron populations, including RGCs (Park et al., 2008), propriospinal neurons (Wu et al., 2015), cortical neurons (Liu et al., 2010; Du et al., 2015) and DRGs (Abe et al., 2010; Christie et al., 2010; Zhou et al., 2012). Here we assessed if a similar strategy aimed at concurrently tackling both intrinsic and extrinsic obstacles has an additive effect and promotes more adult sensory axon regeneration across the DREZ into spinal cord after a dorsal root crush than either approach alone. We transduced adult DRG neurons with caRheb to increase their intrinsic growth capacity after dorsal root injury. Additionally, we injected ChABC into spinal cord dorsal horn to digest upregulated CSPGs at the DREZ. We found this combination did not improve the ability of axons to grow back into spinal cord (though this result may be clouded by intraganglionic inflammation that was induced by the viral injections), but did enhance integration of those axons that did regenerate to form synapses.

## Materials and Methods

### Adeno-Associated Virus (AAV)

AAV5 vectors were obtained and prepared as described before (Wu et al., 2015). All single-stranded AAV5 vectors were obtained from the University of North Carolina’s Gene Therapy Center (Chapel Hill, NC, USA). The expression of GFP and caRheb-FLAG were under the control of a chicken ß-actin promoter driven by a chicken ß-actin promoter.

### *In vitro* Analysis of DRG Neurite Regeneration

Culture plates and coverslips were prepared prior to cell harvesting. Six well plates were coated with poly-L-lysine (0.1 mg/mL; Sigma-Aldrich, Saint Louis, MO, USA). Coverslips with aggrecan-laminin spot gradients were prepared according to a previous protocol (Tom et al., 2004). Briefly, glass coverslips were coated with poly-L-Lysine (0.1 mg/ml) and nitrocellulose. Then four drops of 2 μl aggrecan (0.4 mg/ml aggrecan, Sigma-Aldrich, Saint Louis, MO, USA) were spotted on each coverslip and allowed to air dry. Coverslips were incubated with laminin (10 μg/ml, Sigma-Aldrich, Saint Louis, MO, USA) at 37°C for 6–8 h before cell plating.

Single cell suspensions of DRG neurons were prepared as described previously (Tom et al., 2004). DRGs were harvested from adult Wistar rats (225–250 g, Charles River). After trimming the roots, the DRGs were incubated with collagenase (2000 Units/mL) and neutral protease (25 Units/mL; Worthington Biochemical Corporation, Lakewood, NJ, USA) in HBSS (Gibco, Grand Island, NY, USA), at 37°C for 30 min. The DRGs were rinsed several times with HBSS and then gently triturated in culture media that consisted of Neurobasal-A, B-27, GlutaMax and penicillin/streptomycin (Gibco, Grand Island, NY, USA). After two rounds of low-speed spins (2000 rpm × 2 min), the pellet containing the dissociated DRG neurons was resuspended into culture media at a density of 8000 neurons per milliliter. The cells were plated onto poly-L-lysine coated 6-well plates and incubated with AAV5-caRheb or -GFP (1 × 10^9^ GC/mL). Three days later, the DRG neurons were detached from plates using 0.25% trypsin (Sigma-Aldrich, Saint Louis, MO, USA), rinsed three times with fresh culture media, and re-plated on aggrecan–laminin spot gradient coverslips at a density of 4000 cells per coverslip. Half of the coverslips were also incubated with ChABC (Amsbio; 0.5 U/ml) to digest the aggrecan. Cells detached from six well plates were replated on spot assay coverslips and incubated for 5 days.

After 5 days, the cultures were fixed with 4% PFA in 0.1 M PBS and then processed for immunocytochemistry. The coverslips were rinsed in fresh PBS, incubated in blocking solution (5% normal goat serum, 0.1% BSA, 0.1% Triton X-100 in PBS) for 1 h at room temperature and then in primary antibody diluted in blocking solution overnight at 4°C. The primary antibodies used were anti β tubulin type III (Sigma-Aldrich, 1:1000), anti-FLAG (Sigma-Aldrich 1:500), and anti-GFP (Millipore, 1:500). The next day, the coverslips were rinsed in PBS and then incubated in the appropriate AlexaFluor-conjugated secondary antibody (Invitrogen) for 2 h at room temperature. The coverslips were rinsed in PBS, mounted onto glass slides using FluorSave (EMD Biosciences), and examined using an Olympus BX51 fluorescence microscope.

To quantify the number of neurites crossing the rim, neurites that completely crossed the rim region were counted per spot and normalized to the number of neurons present in the center region of the aggrecan spot. The average number of crossing neurites per spot was calculated per group and normalized to the value of the AAV-GFP + ChABC group. Three independent experiments were conducted. The data were analyzed for statistical relevance using analysis of variance (ANOVA) followed by a *post hoc* Tukey’s test (GraphPad Prism). A *p*-value < 0.05 was considered significant.

### Animals

Adult female Wistar rats from Charles River weighing 150–175 g were used in all experiments. Animals were housed, given unlimited access to food and water, and used in accordance with Drexel University Institutional Animal Care and Use Committee and National Institutes of Health guidelines for experimentation with laboratory animals. Animals were allowed to acclimate for a least 1 week after arrival before any procedure was done.

### Surgical Procedures

#### Dorsal Root Crush Injury

We chose to crush C4-T1 dorsal roots because these roots correspond to the the dermatomes of the majority of the forelimb (Takahashi and Nakajima, 1996) and because this is an established model to assess axon regeneration across a DREZ (Ramer et al., 2001). Animals were anesthetized with isoflurane and kept on a heating pad to prevent hypothermia. Under aseptic technique, the right C4 to T1 DRGs and associated dorsal roots were fully exposed. A small slit was made in the dura immediately caudal to each dorsal root and the tines of a #5 forceps were inserted above and below the root, halfway between the distal end of the dorsal root and the DRG. The forceps were squeezed for 10 s to crush the root. This process was repeated three times to ensure injury completeness.

#### Virus Injection

Similar to what we saw previously (Wu et al., 2015), the expression of the reporter GFP was identified in the soma and axons of transduced neurons, but the expression of the FLAG tag was restricted to the soma. Therefore, we mixed AAV5-GFP with AAV5-caRheb before injection so that we could use GFP to identify the axons of caRheb-expressing neurons. For caRheb-treated animals, 2 μl of AAV5-GFP (8 × 10^9^ GC/μL) and 8 μL of AAV5-caRheb (2 × 10^9^ GC/μL) were mixed (final titer of 1.6 × 10^9^ GC/μL for each vector). For GFP treated animals, 2 μL of AAV5-GFP (8 × 10^9^ GC/μl) was mixed with 8 μL of PBS for a final titer of 1.6 × 10^9^ GC/μl. Immediately after dorsal root crush, a glass micropipette attached to a 5 μL Hamilton syringe was carefully inserted into fully exposed ipsilateral C5–C8 DRGs. One microliter of the mixture of AAV5-GFP and AAV5-caRheb or AAV5-GFP alone was slowly injected into each DRG (0.1 μl AAV5 per min). The glass needle was left in place an additional 1–2 min before removal to prevent reflux.

#### ChABC Injection

Immediately after AAV injections were completed, 1 μl ChABC (50 U/ml) or PBS was slowly injected (0.1 μl per min) into the ipsilateral dorsal horn from C5 to C8 for each rat.

After all surgical procedures, a piece of silastic membrane (BioBrane; UDL Laboratories, Rockford, IL, USA) was placed over the cord and DRGs that has been exposed. The overlying musculature was closed using 5–0 sutures, and the skin was closed using wound clips. Animals were given Lactated Ringer’s, cefazolin (20 mg/kg), and slow-release buprenorphine (0.1 mg/kg; ZooPharm, Windsor, CO, USA) peri-operatively. They were returned to their home cages once they became alert and responsive.

Overall, five groups of animals (GFP + PBS, caRheb + PBS, GFP + ChABC, caRheb + ChABC, an additional control group which only received dorsal root crush) were used in the *in vivo* study. Each experimental group has six animals and the control group has three animals.

### Behavioral Analyses

All animals were habituated to the testing apparatus at least once prior to obtaining baseline scores. Behavioral testing was conducted pre-operatively to establish the baseline responses and then weekly after dorsal root crush injuries on both left (contralateral) and right (ipsilateral) front paws. Paw testing order was determined randomly to minimize an order effect.

#### Hargraeves Test

Changes in thermal sensation after injuries were measured by the Ugo Basile Plantar Heat test (Comerio VA, Italy) as first described by Hargreaves et al. (1988). Briefly, on each testing day, animals were placed in a clear Plexiglass box and allowed to acclimate for at least 10 min. Afterwards, a noxious infrared light beam was applied to the plantar surface of the paw by placing the infrared heat source directly beneath the center of the plantar surface of the paw to be tested. The infrared stimulus application and timer were activated simultaneously. When animals withdrew their paws, the light source turned off and the paw withdrawal latency was recorded in seconds. The infrared stimulus automatically shuts off at 30 s to avoid tissue damage to the paw of any aresponsive animals. Observations of any responses occurred during application of the thermal stimulus, including licking the paw, turning the head to look at the stimulus, or vocalization during stimulation were also recorded. Five trials were performed for each paw with at least 1 min intervals between each trial. The recorded paw withdrawal latencies for each paw were averaged and analyzed for significant differences between groups using a two-way ANOVA and Bonferroni post tests for each time point (GraphPad Prism5). A *p*-value of <0.05 was considered significant.

#### Von Frey Filament Test

The up-down method described by Chaplan et al. (1994) for Von Frey filaments testing was used to measure the degree of tactile sensory changes after dorsal root crush injuries. On each testing day, animals were placed in a metal chamber with a wire mesh bottom and acclimated to the testing environment for at least 10 min. Each trial session began using filament size 4.93 (8 g). If a positive response to a particular filament was observed, the next lower value filament was to be used in the subsequent trial. A negative response resulted in the next higher value filament being used in the subsequent trial. A total of 10 trials were conducted for each paw. The response threshold was the lowest force that produced a positive response, which included paw withdrawal, vocalizing, licking, or guarding in at least 50% of the applications. Similar to the Hargraeves test, the response thresholds of animals in different groups were compared using a two-way ANOVA and Bonferroni post tests for each time point (GraphPad Prism5). A *p*-value of <0.05 was considered significant.

### Electrical Stimulation to Induce c-Fos Expression

One month after dorsal root crush, animals were anesthetized with ketamine and xylazine. The ulnar and median nerves were isolated, dissected free and placed on a bipolar hook electrode for stimulation. The nerves were bathed in a pool of mineral oil to prevent dessication of the nerves throughout the recording period. The nerve was stimulated for 30 min using 1 mA amplitude, 100 μs pulse duration and 50 Hz frequency, similar to what we did previously (Tom et al., 2009, 2013). Animals were perfused 1 h later with 4% PFA and immunofluorescent staining for c-Fos and NeuN were performed on C5–C8 spinal cord sections. The number of c-Fos expression nuclei in spinal cord dorsal horn were counted and compared between groups using a one-way ANOVA and Bonferroni *post hoc* tests.

### Histology

One month after dorsal root crush injury, animals were euthanized with Euthasol and perfused with ice cold 0.9% saline followed by ice cold 4% PFA. Under magnification, the spinal cords from C4 to T1 with roots attached and DRGs from C5 to C8 were dissected out, postfixed in the same fixative overnight at 4°C, and cryoprotected in 30% sucrose for at least 48 h. Tissues were embedded in OCT prior to sectioning using a cryostat. Twenty five micrometer coronal sections of spinal cord tissue were cut and collected serially. DRGs were cryosectioned at a thickness of 20 μm and mounted onto gelatin-coated slides. Sections were blocked in 5% normal goat serum and 10% BSA with 0.1% Triton X-100 in PBS for 1 h. After blocking, sections were incubated with primary antibodies at 4°C overnight. The primary antibodies used were anti-GFP (1:500; Millipore), anti-FLAG (1:1000; Sigma-Aldrich), anti-phosphorylated ribosomal protein S6 (p-S6; 1:800; Cell Signaling), anti-C-4S (1:400, Millipore), anti-calcitonin gene related peptide (CGRP; 1:1000, Peninsula Laboratories International), anti-IB4 (1:1000, Sigma), anti-NF-200 (1:500, Sigma), anti-Iba1 (1:2000, Wako), anti-ED1 (1:200 Millipore), anti-p75 (1:1000 Millipore), and anti-c-Fos (1:1000, Abcam). The next day, sections were washed, incubated with appropriate AlexaFluor-conjugated secondary antibodies (Invitrogen) for 2 h, washed in PBS, mounted onto slides, and coverslipped with FluorSave (EMD Chemical). Stained sections were analyzed on Olympus BX51 and Leica DM5500B epifluorescent microscopes and a Leica TCS SP2 confocal microscope equipped with a Leica DMRE microscope.

### Quantification of p-S6 Expression in AAV Transduced Neurons

To assess numbers of transduced DRG neurons expressing p-S6, DRG sections from DRGs injected with AAV5-GFP (*n* = 3) or the mixture of AAV5-caRheb/AAV5-GFP (*n* = 3) were processed for immunohistochemistry. An additional group of DRG sections from animals that received dorsal root crush but not virus injections were included as an additional control. Every sixth section (120 μm apart) from DRG was immunostained for p-S6 and GFP. All sections were processed at the same time and images for all sections were acquired with the same digital camera and exposure times. Images of naïve DRGs were used to set the threshold to eliminate background noise. Using ImageJ, GFP^+^ and p-S6^+^ neurons whose signal intensities were above the threshold were identified and counted. The percentage of GFP^+^ neurons that also expressed p-S6 was calculated and compared amongst groups using a Chi-square test (GraphPad, Prism5).

### Quantitation of Axons Regenerating Across the DREZ

A subset of coronal spinal cords sections at 750 μm intervals were immunostained for GFP to visualize axons of transduced DRG neurons. We also stained these sections for p75 to visualize Schwann cells within the dorsal root to allow us to definitively determine the boundary of the DREZ. While blinded to treatment group, GFP^+^ axons beyond the p75^+^ labeled CNS/PNS boundary were counted using an Olympus BX51 microscope that had an ocular micrometer. Based on the distance they traveled across the DREZ, axons were binned into 50 μm intervals. The numbers of axons per distance in each section were averaged for each rat subset. The numbers of axons at each length for each treatment group were compared for statistical significance using a one-way ANOVA followed by a Bonferroni correction (*p* < 0.05 criterion for significance; GraphPad Prism5).

## Results

### Expression of caRheb in Adult DRG Neurons Promotes Axon Growth *in vitro*

**Figure 1:**
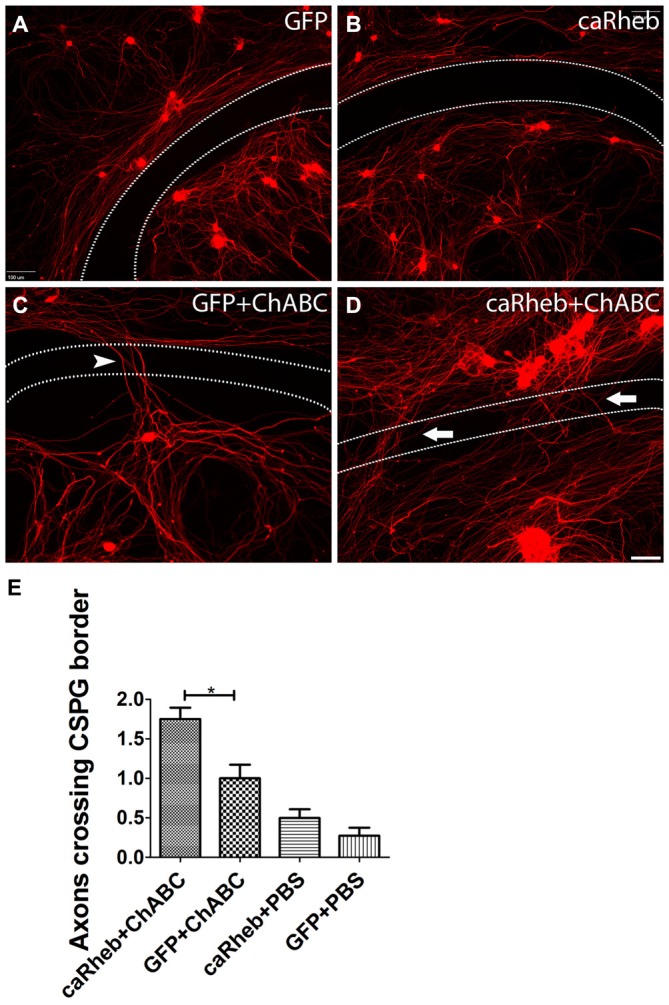
**Combining caRheb expression and chondroitinase ABC (ChABC) promoted neurite crossing of an inhibitory proteoglycan barrier. (A–D)** Representative images of dorsal root ganglion (DRG) cultures are shown. DRG neurons and their neurites were visualized with β-tubulin III staining (red). Neurons transduced with adeno-associated virus (AAV)-GFP **(A)** and AAV-caRheb **(B)** failed to traverse the inhibitory rim (the region between the two dashed lines). ChABC treatment of the aggrecan-containing substrate enabled neurites to cross the inhibitory rim (**C**, arrowhead). Combining ChABC with expression of caRheb in DRG neurons resulted in significantly more robust axon crossing of the rim (**D**, arrows). **(E)** The number of axons crossing the inhibitory rim was counted and averaged in each of the four groups of DRG cultures (*n* = 6 per group). The most crossing was observed in ChABC-treated cultures transduced with AAV-caRheb. *P < 0.05 (one-way ANOVA and *post hoc* Tukey’s tests). Scale bar: 100 μm.

We first sought to determine if activation of mTOR could enhance axonal regeneration on an inhibitory substrate *in vitro* that mimics the environment of the DREZ after injury (Steinmetz et al., 2005). To do so, we plated dissociated, adult DRG neurons on an established *in vitro* model of the glial scar containing the CSPG aggrecan and laminin (Tom et al., 2004). While neurons were able to attach in the center of the CSPG spot, where the concentration of CSPG is low, the rim of the spot has a high concentration of CSPG and a low concentration of laminin, making for an area of marked inhibition for growth (area between dashed lines in Figure 1A). Without any manipulation of the DRG cultures, neurites appeared trapped in the aggrecan spot when they encountered the potently inhibitory rim. Indeed, β-tubulin III labeled neurites from DRGs transduced with AAV-GFP did not cross this rim (Figures 1A,E), similar to the inability of axons to regrow across an untreated DREZ following dorsal root crush. Expressing caRheb alone in the DRGs did not improve neurites’ ability to cross the rim (Figures 1B,E). When the CSPGs in the substrate were digested with ChABC, ~30% of neurites axons were able to cross (arrowhead in Figure 1C), suggesting improved regeneration of a small percentage of sensory axons after cleavage of the inhibitory moieties from the CSPGs. This also suggests that some inhibitory molecules remain within the rim after ChABC, like the CSPG protein core (Imagama et al., 2011). Interestingly, when ChABC is combined with expressing caRheb in DRGs, twice as many axons were able to overcome this lingering inhibition and traverse the rim (arrows in Figures 1D,E; *p* < 0.05). These data suggest that expressing caRheb in adult DRG neurons allows more axons to overcome inhibition that remains after ChABC digestion, resulting in even more regeneration.

### Characterization of DRG Transduction

**Figure 2:**
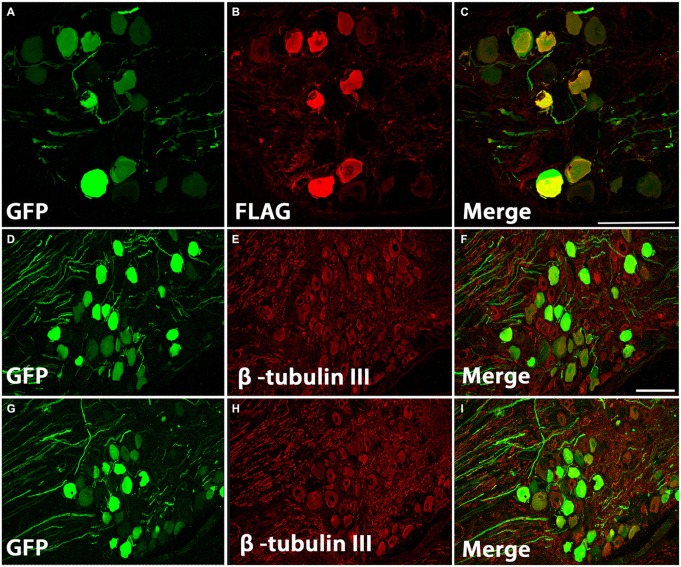
**AAV5 effectively transduced DRG neurons. (A–C)** DRG neurons were transduced with a mixture of AAV-GFP and AAV-caRheb, the latter of which also contained a FLAG tag. One month later, DRGs were stained for GFP **(A)** or FLAG **(B)**. GFP^+^ neurons coexpress FLAG **(C)**, indicating both viruses transduced the same neuron population. **(D–I)** DRGs injected with AAV-GFP **(D–F)** or AAV-GFP/-caRheb **(G–I)** were sectioned and processed for GFP (green) and β -tubulin III (red) staining. In both groups of DRG sections, over 50% of β-tubulin III labeled neuronal cells were also GFP^+^. Scale bar: 100 μm.

To determine if the improved axon growth we observed in the *in vitro* experiments translates to an *in vivo* injury setting, we shifted to a cervical dorsal root crush model. Animals received unilateral intraganglionic injections at C5–C8 of either AAV5-GFP or a mixture of AAV5-GFP/AAV5-caRheb after dorsal root crush. The caRheb vector also contains a FLAG tag. Thus, cells transduced with AAV5-GFP were GFP^+^ and cells transduced with AAV-caRheb were both GFP^+^ and FLAG^+^. We first sought to characterize how efficient our transduction was and which DRG neurons were transduced. We stained DRGs 1 month after injection. We found that injections of AAV-GFP (Figures 2D–F) or -caRheb (Figures 2G–I) into DRGs transduced many DRG neurons, as over 50% β-tubulin III^+^ DRG neurons (Figures 2E,H) co-expressed GFP (Figures 2D,G). Transduction appeared to be neuron specific, as all GFP^+^ cells were also β-tubulin III^+^. Additionally, we found that AAV5-GFP and AAV5-caRheb targeted the same cell population when viruses were mixed and injected together. Neuronal cell bodies expressing GFP (Figures 2A,C) were also labeled with FLAG (Figures 2B,C). Unlike GFP, FLAG was not robustly expressed in axons. Therefore we used GFP to visualize neurons expressing caRheb and their axons in other analyses described below.

**Figure 3:**
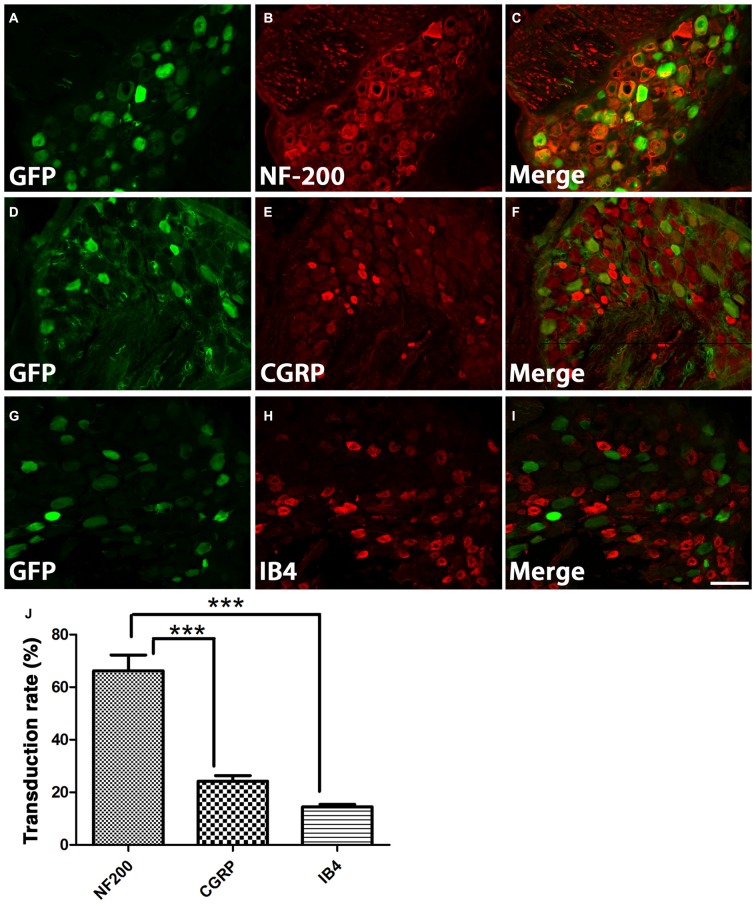
**Characterization of what DRGs subtypes were transduced. (A–I)** DRGs were immunostained for GFP (green) and NF-200 (**B**, red), calcitonin gene related peptide (CGRP; **E**, red), or IB4 (**H**, red) 1 month after AAV injections. GFP expression **(A,D,G)** was mostly observed in NF-200^+^ neurons **(C)**, rather than CGRP^+^ **(F)** or IB4^+^ neurons **(I). (J)** Quantification of transduction efficiencies of AAV5 in NF-200^+^, CGRP^+^ and IB4^+^ DRG neurons revealed that AAV5 has a significantly higher transduction rate for NF-200^+^ neurons, which are large diameter DRG neurons. Sections 120 μm apart from three animals were analyzed. ***P < 0.005 (one-way ANOVA followed by *post hoc* Bonferroni tests). Scale bar: 100 μm.

**Figure 4:**
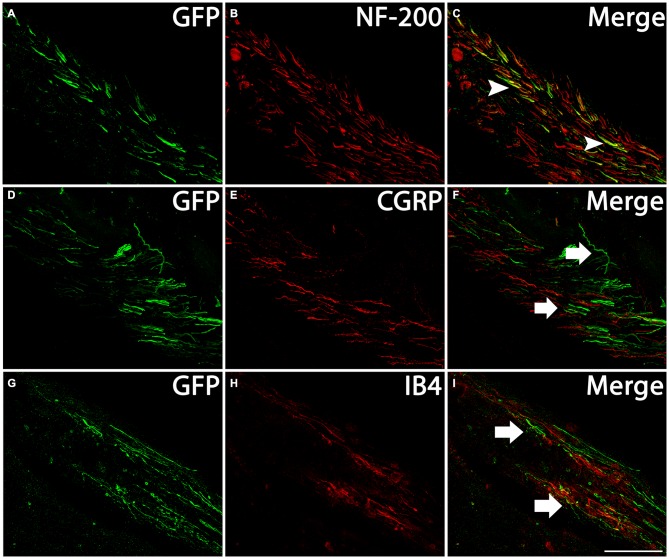
**Profile of axons of transduced neurons in the dorsal root.** Transverse sections of spinal cord at cervical level with the dorsal roots attached were immunostained for GFP (green) and NF-200 (**B**, red), CGRP (**E**, red), or IB4 (**H**, red) 1 month after AAV injections. Many GFP^+^ axons **(A,D,G)** were present in the dorsal root, including near the dorsal root entry zone (DREZ). Merged images indicate GFP-expressing axons coexpressed NF-200 (**C**, arrowheads), but not CGRP (**F**, arrows) or IB4 (**I**, arrows). Scale bar: 100 μm.

DRGs contain several subtypes of sensory neurons that can be generally classified as large diameter, small diameter, peptidergic, or small diameter, nonpeptidergic neurons. We wanted to determine if AAV5 has the same transfection efficiency for all DRG neurons. One month after AAV5-GFP injections, sections from injected DRGs were incubated with antibodies against GFP and NF-200, CGRP or IB4. The vast majority of GFP^+^ neurons (Figures 3A,C) co-stained with NF-200 (Figures 3B,C), a marker for large-diameter neurons. Significantly fewer GFP^+^ neurons were co-stained with CGRP (Figures 3D–F), a marker for the small, peptidergic neurons, or IB4, a marker for small, nonpeptidergic neurons (Figures 3G–I). Quantification of transduction efficiencies of AAV5 in NF-200^+^, CGRP^+^ and IB4^+^ DRG neurons are shown in Figure 3J. Consistent with what we observed in DRG, we detected abundant GFP^+^ axons in the dorsal root 1 month after AAV5 injections. When transverse spinal cord sections containing the dorsal roots were co-stained with antibodies against GFP and NF-200, CGRP or IB4 (Figure 4), GFP^+^ axons were mostly colocalized with NF-200^+^ axons (Figure 4C, arrowheads) rather than CGRP^+^ axons (Figure 4F, arrows) or IB4^+^ axons (Figure 4I, arrows) in the root. These data indicate that mainly large caliber neurons were transduced to express caRheb and/or GFP.

### Expressing caRheb in DRG Neurons Activates Phosphorylation of S6

**Figure 5:**
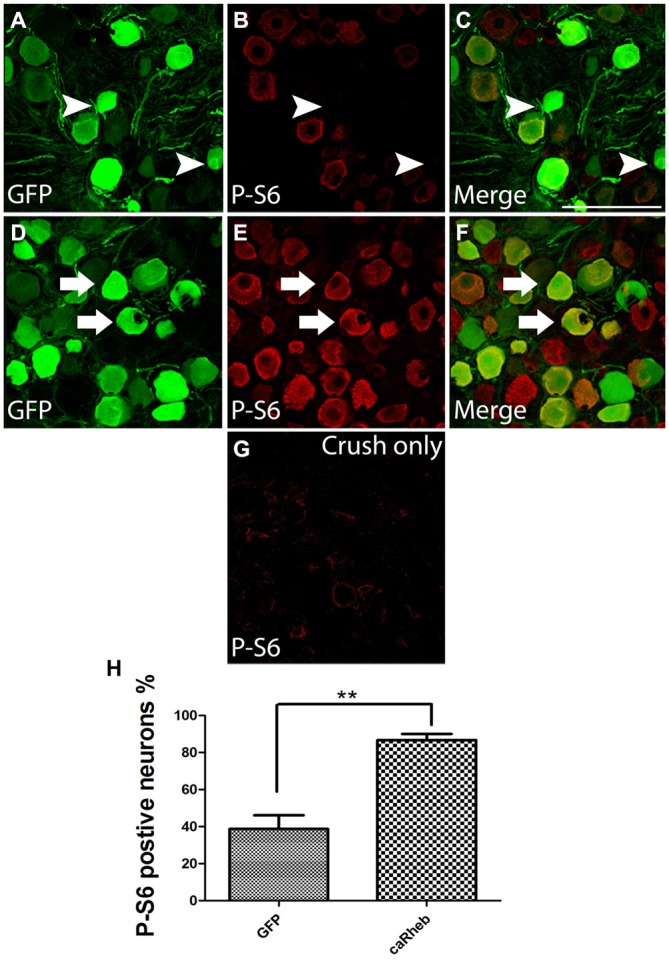
**Expressing caRheb in DRG neurons induced p-S6. (A–F)** DRG sections from rats 1 month after intraganglionic AAV-GFP or AAV-GFP/-caRheb injections were stained for GFP (green) and p-S6 (red). GFP^+^ neurons **(A)** showed low immunoreactivity for p-S6 **(B)**. The merged image confirmed that some GFP^+^ control neurons did not express p-S6 (**C**, arrowheads). CaRheb^+^ neurons **(D)** had significantly higher levels of p-S6 expression (**E,F**, arrows). **(G)** In DRGs from animals that received a dorsal root crush but no virus injection, there was virtually no detectable p-S6. **(H)** Quantification of the AAV-transduced neurons that were also p-S6^+^. AAV-GFP injection induced p-S6 expression in about 40% transduced neurons. Injecting AAV-caRheb increased the number of p-S6 expressing neurons to over 80%. Sections 120 μm apart per animal were analyzed and there were three animals per group, **P < 0.05 (Chi-squared tests). Scale bar: 100 μm.

Rheb-mediated activation of mTOR causes phosphorylation, and thus activation of, S6 ribosomal protein. To provide evidence that the caRheb enhanced activation of S6 in DRGs, we determined the level of phosphorylation of S6 in DRGs from animals 1 month after dorsal root crush and injecting AAV5-GFP (Figures 5A–C) or AAV5-caRheb (Figures 5D–F) via immunohistochemistry for p-S6. DRG sections from animals that received dorsal root crush only without virus injection were also immunostained for p-S6 (Figure 5G). Dorsal root crush alone did not appear to significantly activate S6, as indicated by a virtual absence of any p-S6 immunoreactivity (Figure 5G). We noticed that in the animals that received virus injections, regardless of which vector was used, some neurons (both those that were transduced and those that were not) expressed p-S6 (Figures 5B,E), suggesting that the injection of virus activated S6 to a certain degree. Moreover, we found that expressing caRheb increased this. Around 40% of the GFP-expressing neurons (Figures 5A,C) had some p-S6 immunoreactivity (Figures 5B,C), though most did not (arrowheads in Figures 5A–C,H). On the other hand, strong p-S6 immunoreactivity was detected in more than 80% of caRheb-expressing neurons (arrows in Figures 5D–F,H). These data suggest that virus injection alone activates mTOR but that expressing caRheb in DRG neurons further activates the mTOR pathway.

### ChABC Digests CSPGs at the DREZ

**Figure 6:**
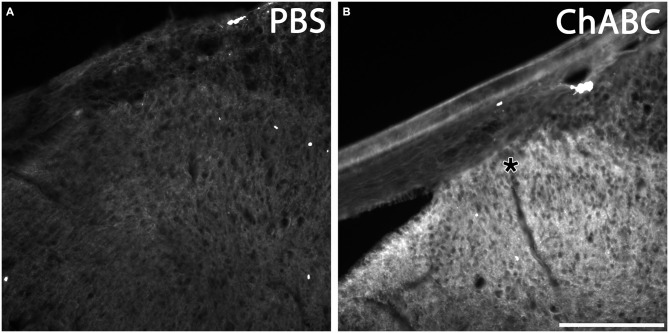
**ChABC digested chondroitin sulfate proteoglycans (CSPG) in spinal cord dorsal horn and DREZ.** Transverse sections of spinal cord with dorsal root from animals 1 month after PBS or ChABC injection into dorsal horn were sectioned and stained with C-4-S, enabling visualization of the sugar stub that remained following ChABC-digestion. After PBS treatment, there was no detectable C-4-S immunoreactivity throughout the spinal cord tissue, including the dorsal root and DREZ **(A)**. In contrast, tissue treated with ChABC **(B)** exhibited high immunoreactivity for C-4-S near the ChABC injection site, including in the dorsal columns, dorsal horn, and the DREZ (asterisk). Scale bar: 200 μm.

It was shown that the bacterial enzyme ChABC delivered via a single *in vivo* microinjection can maintain activity for at least 10 days (Lin et al., 2008). However, we wanted to confirm that a single ChABC injection digested upregulated CSPGs at the DREZ after dorsal root crush. One month after we injected ChABC or PBS into the dorsal horn immediately after dorsal root crush, we incubated sections of spinal cord with the dorsal root attached with the C-4-S antibody to detect the 4-sugar “stub” that remains following ChABC-digestion. PBS treatment failed to produce any digestion of CSPGs in spinal cord and DREZ, as there was virtually no C-4-S staining in these animals (Figure 6A). On the other hand, ChABC injection resulted in widespread C-4-S immunoreactivity (Figure 6B), indicating that one injection of ChABC was effective and resulted in wide-spread digestion of CSPGs, including in the dorsal columns, dorsal horn, and the DREZ (asterisk in Figure 6B).

### Axonal Regeneration at DREZ

**Figure 7:**
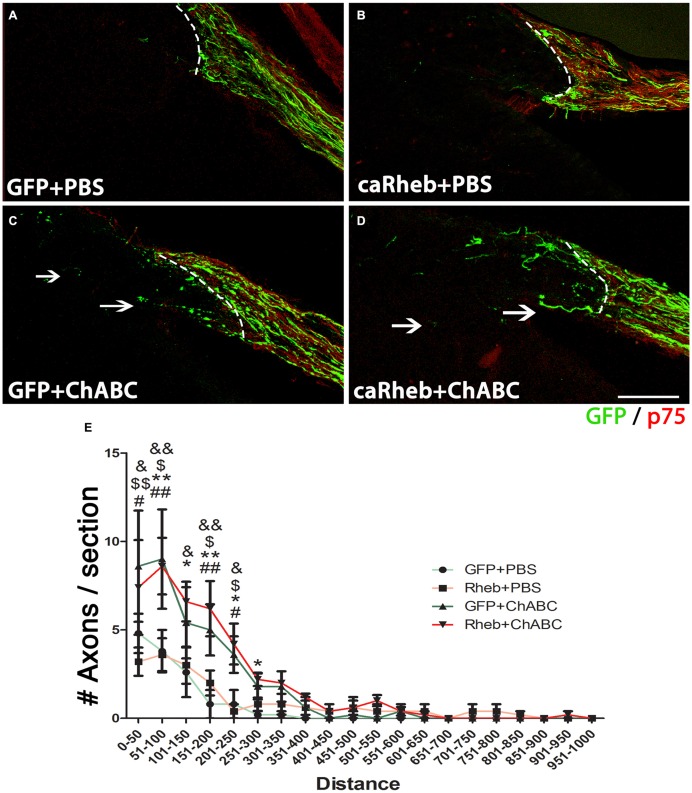
**ChABC treatment enhanced axon regeneration across the DREZ. (A–D)** Transverse spinal cord tissue sections containing the dorsal root and the DREZ (indicated by the dashed line) were immunostained for GFP (green; to visualize axons from transduced DRG neurons) and p75 (red; to visualize Schwann cells within the dorsal root to best determine the boundary between the peripheral nervous system (PNS) and the CNS). Confocal images of the dorsal root and spinal cord of representative sections 1 month after dorsal root crush are shown. Regardless of whether DRGs were transduced with GFP **(A)** or caRheb **(B)**, there was very little to no axon regeneration across a PBS-treated DREZ. CSPG digestion with ChABC improved the ability of axons to traverse the PNS/CNS interface. When ChABC digestion of CSPG at DREZ was combined with AAV-GFP **(C)** or AAV-caRheb **(D)** injection into the DRGs, many more axons were able to extend across the DREZ (arrows). **(E)** Regenerating GFP^+^ axons beyond the DREZ were counted in a subset of sections and binned into four groups based on the distance distal to the DREZ boundary. Significantly more axons grew short distances (0–300 μm) across the DREZ in both ChABC-treated groups than in both PBS-treated groups. Expressing caRheb did not enhance this ChABC-facilitated regeneration. There were six animals per group and five sections per animal were analyzed. ^#^*P* < 0.05, ^##^*P* < 0.01 GFP + PBS vs. GFP + ChABC; *P < 0.05, **P < 0.01 GFP + PBS vs. caRheb + ChABC; ^$^*P* < 0.05, ^$$^*P* < 0.01, caRheb + PBS vs. GFP + ChABC; ^&^*P* < 0.05, ^&&^*P* < 0.01, caRheb + PBS vs. caRheb + ChABC (one-way ANOVA followed by *post hoc* Bonferroni tests). Scale bar: 200 μm.

It has been demonstrated that ChABC-mediated digestion of glycosaminoglycan chains on CSPGs can promote axonal regeneration, including at the DREZ (Cafferty et al., 2007, 2008; Tom et al., 2009; Cheng et al., 2015). Here we assesssed whether expressing caRheb in DRG would allow more axons to extend beyond a ChABC-treated DREZ. One month after dorsal root injury, very few GFP^+^ axons were found to have extended beyond a PBS-treated DREZ boundary in AAV5-GFP + PBS treated animals (Figure 7A). Similar to what we observed with descending CNS axons (Wu et al., 2015), expressing caRheb in neurons while leaving the glial scar intact did not improve sensory axons’ ability to extend across the inhibitory environment of the DREZ (Figure 7B). Almost all axons failed to regenerate past the DREZ. Modification of the scar with ChABC did improve regeneration (Figures 7C,D), as shown previously. ChABC treatment significantly increased the number of axons that grew beyond the DREZ (Figure 7E). While axons were observed to penetrate the DREZ, the majority traveled only small distances across the DREZ. When we combined AAV-caRheb microinjection into the DRG along with ChABC microinjection into the spinal cord dorsal horn, a similar number of GFP^+^ regenerating axons were found across the DREZ. These data indicate that ChABC increased the number of axons regenerating through DREZ and expressing caRheb in DRGs did not further enhance this growth response.

### Behavioral Analysis

**Figure 8:**
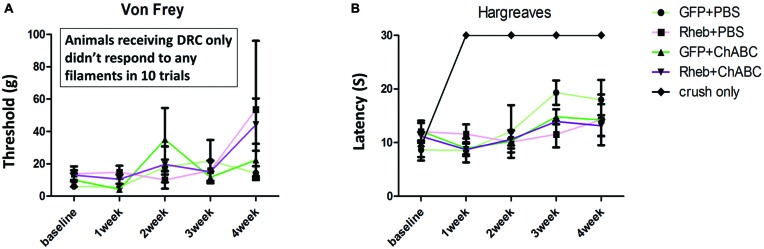
**All AAV-injected animals “responded” to sensory stimuli.** Five groups of animals (GFP + PBS, caRheb + PBS, GFP + ChABC, caRheb + ChABC, an additional control group which only received dorsal root crush) were tested using the Von Frey filament and Hargreaves tests before surgery and weekly after surgery. As expected, dorsal root crush only animals were aresponsive to mechanical **(A)** or thermal **(B)** stimuli. Interestingly, all animals with AAV injections responded to both forms of sensory stimuli. No differences between the AAV groups were observed in either test at any time point (*N* = 6, two-way ANOVA).

To determine if treatment with ChABC or ChABC and AAV5-caRheb promoted any functional improvement after dorsal root crush, sensory function of the animals was assessed using the Hargreaves test to examine thermal sensation and the Von Frey filament test to examine fine touch sensation. Because the animals did not place their injured paws down during the first week after surgery, the earliest time point for both behavioral tests was 1 week post injury. Interestingly, all animals that received AAV injections were responsive to both mechanical (Figure 8A) and thermal stimuli (Figure 8B). Furthermore, all AAV animals, even the PBS-treated animals that did not have any afferent regeneration (Figure 7), responded similarly—there was no significant difference between any of the treatment groups at any time point. In comparison, animals that received dorsal root crush only (and no intraganglionic injections of AAV) did not respond in either sensory test at any testing point, suggesting that our surgical technique resulted in complete lesions that interrupted afferent input. Thus, intraganglionic injections after a complete dorsal root crush somehow resulted in a sensory “response” that was independent of any regeneration.

### Intraganglionic Injections Activate Macrophages/Microglia

**Figure 9:**
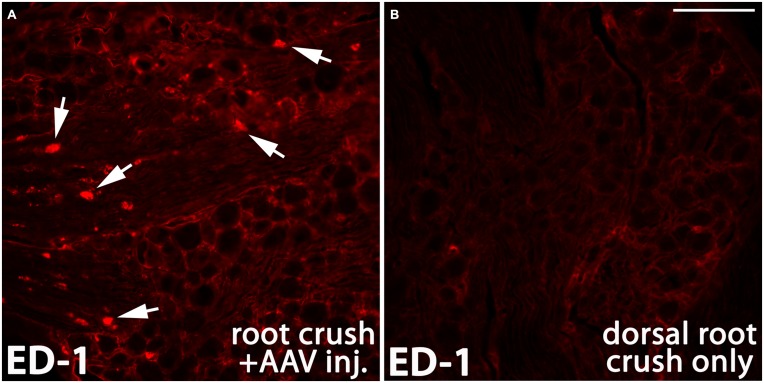
**AAV injection induced invasion of macrophages into the DRG.** One month after AAV injection, ED-1 positive macrophages (arrows in **A**, red) were identified in the DRG. In the control DRGs that did not receive AAV injections, there was very little immunoreactivity for ED-1 **(B)**. Scale bar: 100 μm.

**Figure 10:**
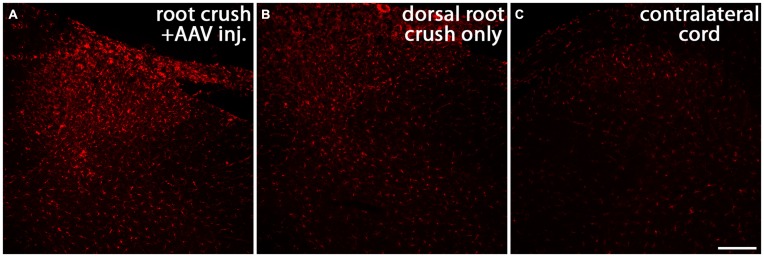
**Both dorsal root crush and intraganglionic AAV injections activate microglia in spinal cord.** Cervical spinal cords were collected 1 month after animals received dorsal root crush only **(B)** or dorsal root crush and virus injection into the DRG **(A)**. Microglia in spinal cord sections were labeled with Iba-1 (red). Strong Iba-1 immunoreactivity was detected in reactive microglia near the DREZ and in the ipsilateral dorsal horn in animals that received both dorsal root crush and AAV injection **(A)**. The immunoreactivity for Iba-1 was significantly lower in animals that only received dorsal root crush **(B)**. There was very little Iba-1 staining in the contralateral dorsal horn **(C)**. Scale bar: 100 μm.

There is compelling evidence indicating that the activity of macrophages/microglia play an important role in initiating neuropathic pain (Detloff et al., 2008; Richter et al., 2012; Segond von Banchet et al., 2013; McDonald et al., 2014; Kobayashi et al., 2015). Moreover, some pro-inflammatory cytokines have been shown to augment neurite outgrowth from injured sensory neurons (Bastien and Lacroix, 2014;Osório et al., 2014; Habash et al., 2015). Thus, we examined whether macrophages invaded the DRGs in animals that were injected with AAV. We noticed that 1 month after AAV injection, an influx of ED-1-positive macrophages can be identified in the DRG (arrows in Figure 9A). No detectable ED-1 was observed in DRGs from animals that received dorsal root crush only and no AAV injections (Figure 9B), indicating that this invasion of macrophages into the DRG was triggered by virus injections. We also wanted to identify whether dorsal root injury and virus injections trigger an inflammatory response in the cervical spinal cord. Iba-1 was used to identify activated microglia in spinal cord sections collected from dorsal root crush only animals (Figure 10B) and animals that received dorsal root crush and AAV5 injections into the DRGs (Figure 10A). Strong Iba-1 immunoreactivity was detected in the spinal cord, especially near the DREZ and dorsal horn, from animals that received dorsal root crush and virus injections (Figure 10A). Immunoreactivity for Iba-1 in animals that only received dorsal root crush was markedly lower (Figure 10B). Moreover, the contralateral side of the spinal cord, showed minimum immunoreactivity for Iba-1 (Figure 10C). The striking differences in levels of Iba-1 between groups indicate dorsal root injury induced the activation of microglia in dorsal horn ipsilateral to the injury and that this inflammatory response was further augmented by injury to the DRG caused by virus injections.

### caRheb Enhances Integration of Regenerated Axons

**Figure 11:**
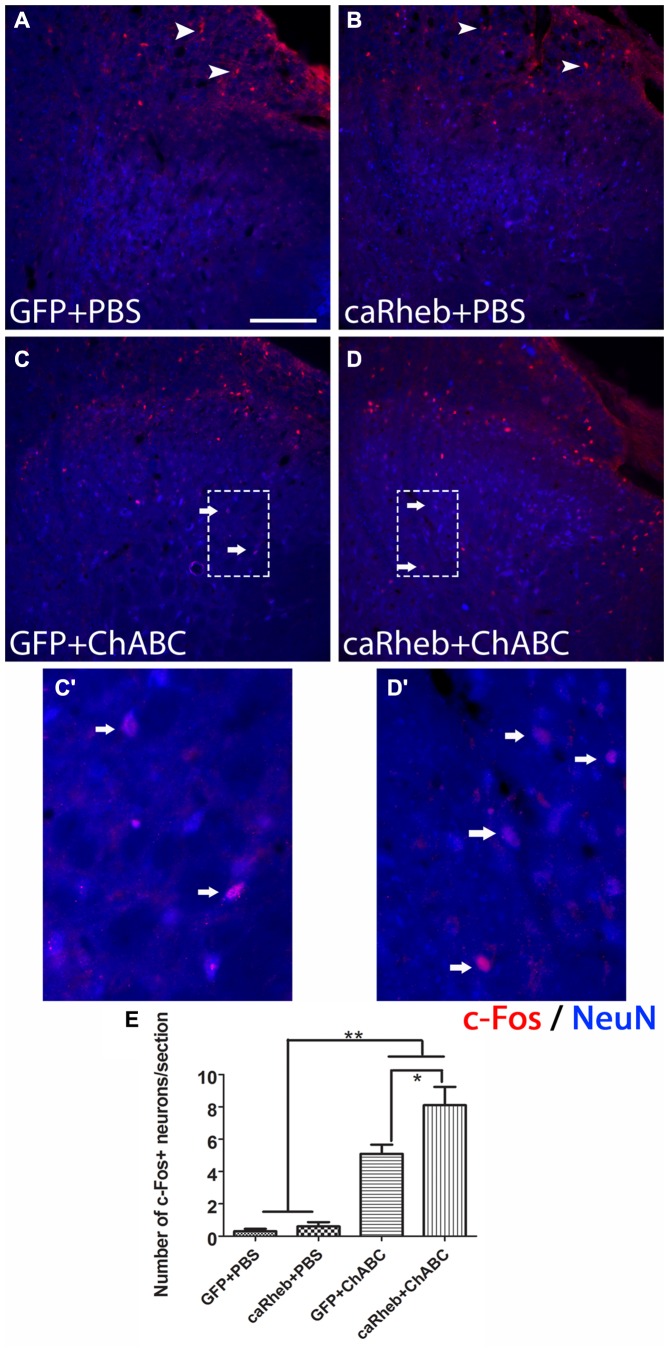
**Expressing caRheb increased the integration of axons that regenerate across the DREZ.** One month after injury, median and ulnar nerves were electrically stimulated for 30 min and animals were sacrificed 1 h later. **(A–D)** Transverse sections of cervical spinal cords were sectioned and processed for c-Fos (red) and NeuN (blue) immunohistochemistry (Lavdas et al., 1997). Animals that were treated with GFP + PBS **(A)** or caRheb + PBS **(B)** had very few c-Fos^+^ nuclei in spinal cord gray matter. Almost all neuronal c-Fos staining was detected in the dorsal root (**A,B**, arrowheads). On the contrary, in both groups of animals treated with ChABC, significantly more c-Fos^+^ neuronal nuclei were detected in gray matter (**C,D**, arrows). High magnification images of the boxed regions in **(C,D)** are depicted in **(C’,D’)**, respectively. **(E)** c-Fos^+^ neuronal nuclei in spinal gray matter were counted and compared. We found that electrical stimulation in caRheb + ChABC animals induced *c-Fos* in the most neurons. Scale bar: 100 μm. *N* = 6. *P < 0.05, **P < 0.01 (one-way ANOVA followed by *post hoc* Bonferroni tests).

We previously showed that when we combine caRheb expression with ChABC treatment, CNS axons can regrow beyond a distal peripheral nerve graft interface to extend into gray matter of host spinal cord tissue and form putative synapses upon host neurons (Wu et al., 2015). Here, we determined whether the afferents that regenerated back into spinal cord formed functional synapses. One month after injury, we electrically stimulated the ulnar and median nerves ipsilateral to the injury side prior to perfusion and performed histological analysis for c-Fos induction in neurons located in the dorsal horn. In both groups of animals that were treated with PBS [AAV-GFP (Figure 11A); AAV-caRheb (Figure 11B)], we saw very few c-Fos^+^ neurons in ipsilateral gray matter. The c-Fos^+^ immunoreactivity was observed mainly in the root, proximal to the DREZ, where the axons stopped elongation. On the other hand, in animals treated with ChABC, significantly more c-Fos^+^ neurons were identified in ipsilateral dorsal horn (Figures 11C,D; arrows). Interestingly, despite there being similar numbers of axon extending across the DREZ in both groups of ChABC-treated animals (Figure 7), stimulation of the median and ulnar nerves in animals treated with ChABC and AAV-caRheb (Figures 11D,D’,E) induced c-Fos in significantly more neurons than in animals treated with ChABC and AAV-GFP (Figures 11C,C’,E). Thus, caRheb expression appears to enhance synaptic formation and/or function of axons that regenerated back into spinal cord gray matter.

## Discussion

Axon regeneration of the peripherally-projecting DRG axon branch after injury to peripheral nerves can be successful. After a dorsal root crush that results in injury to the centrally-projecting branch, almost all axons can grow across the lesion site, extend along the root, and arrive at the DREZ. However, their regeneration ceases at the DREZ and axons remain in the PNS/CNS transitional zone (Di Maio et al., 2011), demonstrating that the DREZ is a potent barrier for axonal regeneration. Both oligodendrocyte-associated inhibitors and astrocyte-associated inhibitors present there cause growth cone collapse and axon retraction, repelling axons from entering the CNS (Golding et al., 1997, 1999). Moreover, Schwann cells in the dorsal root that provide a highly growth-promoting ensheathment do not intermingle with astrocytes in the spinal cord, constraining the extension of axons into CNS territory (Grimpe et al., 2005; Afshari et al., 2010a,b). Neutralization of the inhibitory molecules such as CSPG (Cafferty et al., 2007) or Nogo (Harvey et al., 2009) at the DREZ or increasing levels of neurotrophic factors, such as NGF (Ramer et al., 2000; Romero et al., 2000, 2001), GDNF (Ramer et al., 2000; Harvey et al., 2010) or NT-3 (Ramer et al., 2000, 2002), in the dorsal horn have been partially effective in luring the injured sensory axons back into the spinal cord.

Limited intrinsic growth capacity also contributes to the regeneration failure at the DREZ. Whereas peripheral nerve injury triggers high expression of regeneration associated genes, fostering regeneration of injured peripherally-projecting DRG axons, injury to the dorsal root fails to elicit a similar pro-regenerative gene expression profile (Mason et al., 2002; Seijffers et al., 2006). Peripheral conditioning lesions have been used to enhance the intrinsic regeneration capacity of the centrally-projecting axon after a spinal cord injury (Neumann and Woolf, 1999) or dorsal root injury (Zhang et al., 2007; Di Maio et al., 2011) with some degree of success. Thus, it appears that it is possible to manipulate the intrinsic potential for growth of the central branch. However, even with a conditioning lesion, the growth response of the central branch is still less than that of the peripheral branch. This difference has been attributed to decreased levels of local protein synthesis in central axons vs. PN axons. PNS axons have a high growth capacity with a high content of locally generated proteins that contribute to regenerative growth whereas CNS axons in the adult animals have very low level of translational machinery and low intra-axonal protein synthesis (Verma et al., 2005; Kalinski et al., 2015).

In the current study, we tested the efficacy of a strategy aimed at both activation of intrinsic axon growth capacity (i.e., caRheb-mediated) and digestion of upregulated inhibitory molecules with ChABC to promote sensory axon regrowth across the DREZ. mTOR is a serine/threonine protein kinase expressed in the mammalian nervous system that plays a critical role in protein synthesis (Liang et al., 2013). Importantly, mTOR and its downstream effectors, such as p-S6K, pS6 and p-4EBP1 are found in DRG somas and axons (Verma et al., 2005; Jiménez-Díaz et al., 2008; Liang et al., 2013). The activation of mTOR and its downstream effectors are implicated in the control of growth cone dynamics and guidance during development and axon regeneration after injury (Campbell and Holt, 2001; Nie et al., 2010). Activation of mTOR also promotes compensatory axonal sprouting after CNS injuries (Lee et al., 2014). Furthermore, its activation enhances axon growth capacity in different neuron types (Verma et al., 2005; Park et al., 2008; Liu et al., 2010). Interestingly, it has also been reported that although mTOR is expressed in adult DRGs, its phosphorylated form, which activates its downstream effectors, is expressed at a very low level under normal conditions (Xu et al., 2010). Injuries to peripheral nerve transiently activate mTOR. This activation appears to be important for regeneration as blocking mTOR activity pharmacologically with rapamycin reduces axon growth ability following peripheral injury (Abe et al., 2010; Melemedjian et al., 2011). These observations indicate that protein synthesis mediated by mTOR signaling regulation plays a critical role in promoting sensory axon regeneration. However, what was still unclear is if directly activating mTOR in DRG neurons without injuring the PNS will affect the central branch’s ability to regenerate into the CNS.

In recent years, activation of mTOR has been achieved by genetically silencing its negative regulators, such as tuberous sclerosis complex (TSC) or PTEN (Park et al., 2008; Abe et al., 2010; Liu et al., 2010). In our study, we expressed caRheb, a mutant form of Rheb in DRG, which can directly and constitutively activate mTOR (Kim et al., 2011, 2012). We found that stimulation of the intrinsic growth capacity by caRheb increased mTOR activation was not robust enough to foster regeneration of DRG axons across inhibitory environments, either in an *in vitro* model of glial scar (Tom et al., 2004) or *in vivo* across the DREZ following a dorsal root crush. Manipulation of the inhibitory environment with ChABC *in vitro* augments this growth such that the combination of caRheb with ChABC resulted in more neuritic growth than either treatment alone. However, this was not observed *in vivo*. Combining AAV-caRheb injection in DRG with ChABC treatment of the dorsal horn did not have an additive effect on inducing more axons to grow beyond the DREZ.

The discrepancy between the effects of caRheb + ChABC *in vitro* and *in vivo* might be explained by the more complex, inhospitable environment that the regenerating axons encounter *in vivo*. At the DREZ, both CSPGs and oligodendrocyte-derived inhibitors constrain regeneration (Ramer et al., 2001). In our *in vivo* study, these inhibitors existed and remained intact. The difference in *in vitro* and *in vivo* DRG neuron transduction rates with AAV-caRheb may also contribute to the disparity in the regeneration capacity. In DRG cultures, over 80% DRG neurons were transduced by AAV-caRheb and AAV-GFP. There was no apparent variance in the transduction rate between different DRG neuron subtypes. On the other hand, in animals with AAV injections into DRGs, the overall transduction rate was around 50% and AAV mostly transduced large diameter DRG neurons rather than small diameter neurons. This observation conflicts with findings from a previous study that found that AAV5 transduces small diameter DRG neurons (Mason et al., 2010). The inconsistency between that study and the present one may be explained by differences in the promoter. In that study, GFP expression was under the control of CMV while here, transgene expression was driven by CBA. It is possible that the capacity for regrowth varies greatly among different population of DRG neurons. Indeed, large caliber, myelinated axons are thought to regenerate more poorly than those of small, non-myelinated axons projecting from small DRG neurons (Guseva and Chelyshev, 2006; Di Maio et al., 2011). Therefore, in our *in vivo* study, we examined the regeneration of a subpopulation of axons whose propensity for regeneration is particularly weak.

Another possible explanation between the disparity between the *in vitro* and the *in vivo* data is that injecting AAV intraganglionically appeared to induce p-S6 expression and inflammation in the DRGs. Though p-S6 levels in the caRheb-treated animals was significantly stronger than in the GFP-treated ones, even injecting just AAV-GFP increased the level of the activated form of the mTOR downstream effector p-S6 compared to DRGs from animals that received dorsal root crush only and no virus injections (Figure 5). We speculate that the activation of mTOR in AAV-GFP animals resulted from injection-induced inflammation, as we see an influx of ED-1^+^, activated macrophages into these DRGs. Inflammation, including the recruitment of macrophages into the DRG (Lu and Richardson, 1991; Steinmetz et al., 2005; Kwon et al., 2013) has long been associated with promoting a growth state (Gensel and Zhang, 2015). Interestingly, it has been reported that complete Freund’s adjuvant (Wieseler et al., 2010) induced inflammation significantly increases the activity of mTOR and S6k1 in DRGs and spinal cord dorsal horn (Liang et al., 2013). Thus, it is possible that mTOR plays a role in inflammation-enhanced regeneration.

This potential mechanism has been more extensively studied in RGCs injury models. An inflammatory stimulus induced by injury or by application of mediators of inflammatory stimulation, such as astrocyte-derived ciliary neurotrophic factor (CNTF), leukemia inhibitory factor (LIF) and interleukin-6 (IL-6), or the yeast cell wall component zymosan produce neuroprotective and axon growth stimulating effects (Leon et al., 2000; Yin et al., 2003, 2006; Müller et al., 2007; Leibinger et al., 2012, 2013). The activation of JAK/STAT3 and PI3K/AKT/mTOR signaling cascades were shown to contribute to how an inflammatory stimulus pushes RGCs into a regenerative state (Leibinger et al., 2012). It is possible that inflammation can have a similar effect on DRG neurons and may help to explain the similar extent of anatomical regeneration we observed in the GFP + ChABC and caRheb + ChABC animals. Virus injection inevitably caused some trauma and triggered the infiltration of macrophages into the ganglia. For reasons discussed above, this inflammation may have activated mTOR to levels sufficient to enhance the regenerative capacity.

Whether ChABC digestion alone promotes sensory regeneration is unclear. While some reported that CSPG digestion by ChABC promotes regeneration of the dorsal columns (Bradbury et al., 2002; Grimpe et al., 2005) and transgenic expression of ChABC under the GFAP promoter in mice promotes rhizotomized axons to grow back into spinal cord (Cafferty et al., 2007), there are also reports showing that ChABC treatment alone only allows for a negligible amount of regeneration of sensory axons across DREZ after dorsal root crush in rats (Steinmetz et al., 2005; Cafferty et al., 2008). Our study does not contradict these previous studies. Similar to what was demonstrated previously with zymosan (Steinmetz et al., 2005), inflammation induced by injecting AAV-GFP or AAV-caRheb intraganglionically in our study may have enhanced the effect of ChABC treatment, increasing the number of axons extending across the DREZ.

The injection-induced inflammatory response may also help to explain the inconclusive behavioral data we obtained in the Hargraves and Von Frey behavioral tests. We do not believe that the behavior we observed was mediated by regeneration, as even the PBS-treated animals that did not display any regeneration had “responses” to the sensory stimuli. Rather, we posit that the “responses” were indicative of some form of neuropathic pain. In support of this, the animals with root crushes and AAV injections had considerably more activated microglia within the dorsal horn than the crush-only animals (Figure 10). Furthermore, the extent of activated microglia within the dorsal horn has been shown to be predictive of the development and the extent of neuropathic pain (Detloff et al., 2008). Thus, we may have failed to observe any differences in the responses to thermal and mechanical stimuli between groups because intraspinal inflammation was triggered in all treatment groups.

Although we did not observe any behavioral differences between groups, we did find that regenerated axons in the caRheb + ChABC animals formed significantly more functional synapses on dorsal horn neurons than in GFP + ChABC animals (as indicated by the induction of c-Fos expression in dorsal horn neurons upon the stimulation of ipsilateral median and ulnar nerves). This indicates mTOR activation enhances the integration of sensory axons that regenerate back into spinal cord. Interestingly, mTOR activity is associated with various aspects of excitatory and inhibitory synaptic function, including increasing synaptic strength and affecting synapse number and synaptic vesicle number. Studies have demonstrated that activation of mTOR by PTEN knockdown *in vivo* results in excitatory synapse formation with granule cells (Luikart et al., 2011). Likewise, inhibition of mTOR with rampamycin blocks excitatory synaptic output of cultured dentate neurons (Weston et al., 2012) and reactive excitatory synaptogenesis in the brain after epilepsy-induced synaptic reorganization (Yamawaki et al., 2015). Thus, it has been suggested that mTOR-mediated protein synthesis may participate in synaptogenesis, maintaining synaptic homeostasis, and synaptic output (Lyu et al., 2013). The specific molecular mechanisms underlying mTOR’s role in these activities remain unclear.

In conclusion, we confirmed that caRheb expression effectively activates the mTOR signaling pathway in DRGs and this mTOR activation can promote sensory axon regeneration when combined with CSPG digestion by ChABC *in vitro*. Combining caRheb expression with ChABC digestion of CSPG did not promote more axonal regeneration across the DREZ compared to what was observed with ChABC treatment alone *in vivo*, though this may be attributed to intraganglionic inflammation. However, expressing caRheb did enhance the integration of these regenerated axons with spinal neurons. Even though the underlying mechanisms of mTOR mediated axonal regeneration and synaptogenesis remain to be determined, our data suggest that increasing mTOR level has the potential to facilitate regenerating axons to form synapses with the appropriately-located target neurons. Therefore, this combined treatment has the potential to promote functional recovery after dorsal root injury.

## Author Contributions

DW and VJT designed the experiments. DW, MCK and M-PC performed the experiments. NK and REB provided constructs for transduction. MRD provided technical expertise and assisted with data interpretation. DW and MCK analyzed the data. DW and VJT prepared the manuscript.

## Conflict of Interest Statement

The authors declare that the research was conducted in the absence of any commercial or financial relationships that could be construed as a potential conflict of interest.
